# A unique presentation of infratemporal fossa giant cell granuloma: A case report and literature review

**DOI:** 10.1016/j.ijscr.2025.110851

**Published:** 2025-01-06

**Authors:** Hussain J. Aljubran, Maria R. Alabdulaal, Ali A. Alnasser, Ghadeer F. Bu Saeed, Zahraa J. Almuhanna, Ali Almomen

**Affiliations:** a College of Medicine, Imam Abdulrahman Bin Faisal University, Dammam, Saudi Arabia; b College of Medicine, Arabian Gulf University, Manama, Bahrain; c College of Medicine, King Faisal University, Al-Ahsa, Saudi Arabia; d Department of Otolaryngology Head and Neck Surgery, King Fahad University Hospital, Al-Khobar, Saudi Arabia; e Department of Otolaryngology Head and Neck Surgery, King Fahad Specialist Hospital, Dammam, Saudi Arabia

## Abstract

**Introduction:**

The infratemporal fossa (ITF) is considered an uncommon location for giant cell granuloma (GCG), a rare benign disease that is frequently detected in the maxilla and mandible.

**Presentation of case:**

A 47-year-old male presented with right-sided hearing loss, tinnitus, and jaw claudication. Radiological imaging confirmed the presence of a mass in the ITF accompanied by bone erosion. An endonasal endoscopic approach was used to remove the lesion after a CT-guided biopsy verified GCG. The final diagnosis was histopathologically confirmed.

**Discussion:**

GCG is one of the subtypes of giant cell-rich bone lesions, commonly reported in the anterior or posterior mandible and anterior maxilla. Histological evaluation to distinguish GCG from other giant cell-rich lesions, such as giant cell tumors, particularly in uncommon extragnathic locations like the ITF, is a crucial step after imaging for an accurate diagnosis and management.

**Conclusion:**

In GCG, early identification and surgical excision are essential to avoid complications. This case demonstrates the usefulness of CT-guided biopsy and imaging in detecting GCG in an unusual location.

## Highlights

• Infratemporal fossa has been associated with multiple disease processes.
• Giant cell granuloma has rarely been reported to occur in infratemporal fossa.
• Differentiating between giant cell granuloma and giant cell tumor can be challenging.

## 1 Introduction

Giant cell granuloma (GCG) is a rare, benign, non-neoplastic lesion that involves a reparative process within the bone [1]. GCG has been described as a condition with a locally reparative process in response to hemorrhage, local trauma, or inflammation, although its exact etiology remains unclear. Initially, this condition was referred to as giant cell reparative granuloma. However, recent evidence suggests that the clinical behavior of these lesions differs from a typical reparative reaction. This is because GCG does not resolve or heal on its own and requires treatment or removal, leading to the discontinuation of the term “reparative” in its nomenclature [1]. The potential for recurrence is the primary concern with GCG, with recurrence rates as high as 49 % in surgically treated cases [2]. Since 1953, GCG has been documented in various locations, most commonly in the mandible and maxilla, particularly in younger individuals (20–40 years) [3,4].

Infratemporal fossa (ITF) is adjacent to the base of the skull, bounded superiorly by the greater wing of the sphenoid bone, laterally by the ascending ramus of the mandible, anteriorly by the maxilla, medially by the pterygoid processes, and posteriorly and inferiorly by soft tissues [5]. Various tumor subtypes have been associated with lesions in the ITF. However, no previous studies have reported the occurrence of GCG in this area. Therefore, the purpose of this study is to present a case of a 47-year-old male with a right ITF lesion, ultimately diagnosed as GCG. This case has been reported according to the SCARE guidelines for surgical case reporting. [6]

## 2 Presentation of case

**Fig. 1:**
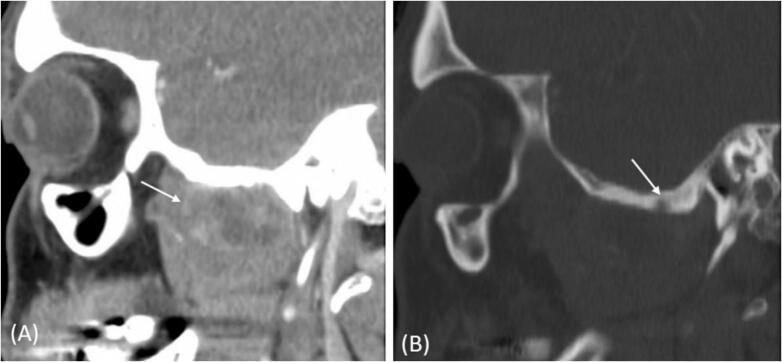
(A) CT scan post contrast with sagittal view in soft tissue reconstruction shows the soft tissue mass (arrow) in right ITF with heterogeneous low density and faint peripheral calcifications. (B) CT scan with sagittal view in bone algorithm reconstruction shows the sclerosis of the temporal bone with erosion of the outer cortex (arrow).

**Fig. 2:**
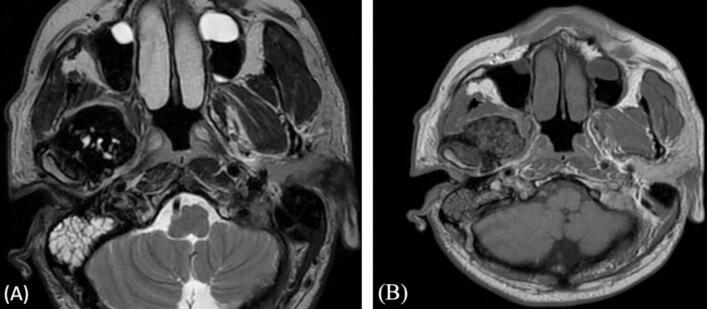
(A) MRI with transaxial T2 view shows the ITF mass with predominant hypointense signal and scattered hyperintense small foci displacing the right masticator space anteriorly up to the posterior wall of the right maxillary antrum. (B) Pre and post contrast axial T1 image shows heterogeneous signal intensity with inherent bright spots and no post contrast enhancement.

A previously healthy 47-year-old male presented to the otolaryngology clinic complaining of deafness, tinnitus, blockage sensation in the right ear, and jaw claudication in the right side for one-year duration. However, the patient had no history of nasal or neurological complaints. Otolaryngological examination showed a swelling in the right peri-auricular area, measuring 2 × 2 cm in size, rubbery, regular, rigid, deep, and not mobile or tender. The ears examination showed dull tympanic membrane in the right side with normal tympanic membrane in the left side. The rest of otolaryngological examination was unremarkable with no palpable cervical lymph nodes. The patient's computed tomography (CT) scan of paranasal sinuses showed heterogeneous mass in the right ITF with soft tissue density and thin peripheral rim of calcifications (Fig. 1A). The mass is inseparable from the inferior surface of the right temporal and sphenoid bones. It results in sclerosis and erosion of the outer cortex of the bones (Fig. 1B). Additionally, the mass displaces the right mandibular condyle posteriorly. Magnetic resonance imaging (MRI) revealed a well-defined mass in the right ITF, inseparable from the floor of the skull base. It measures 20 × 3.5 × 4.4 cm (craniocaudal X anteroposterior X transverse). The mass demonstrates low signal intensity on T1 and T2 with no enhancing soft tissue component (Fig. 2A, B). The mass displaces the lateral pterygoid muscle anteroinferiorly and the parapharyngeal space posteromedially with no intracranial extension or dural involvement.

**Fig. 3:**
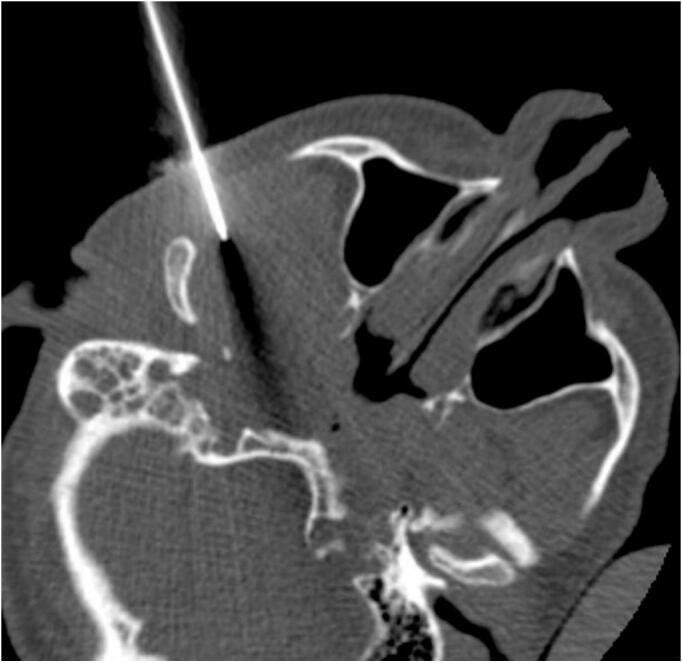
CT-guided percutaneous true-cut biopsy of the right ITF mass.

**Fig. 4:**
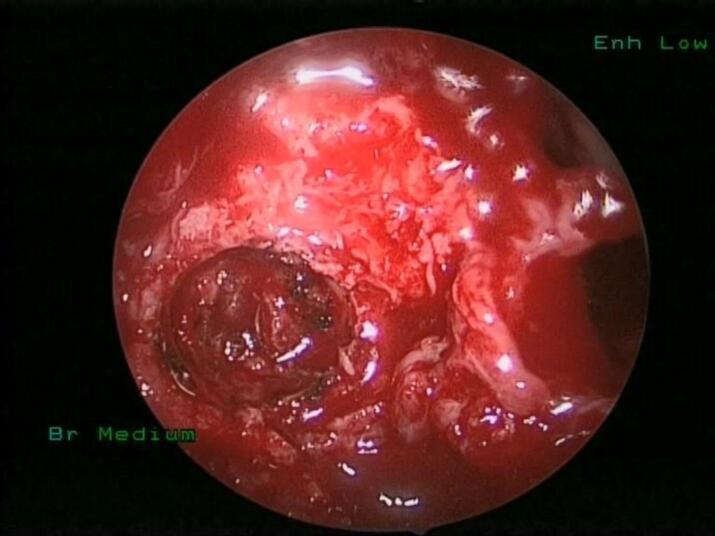
Tumor exposure after removal of posterior maxillary sinus wall (arrow).

**Fig. 5:**
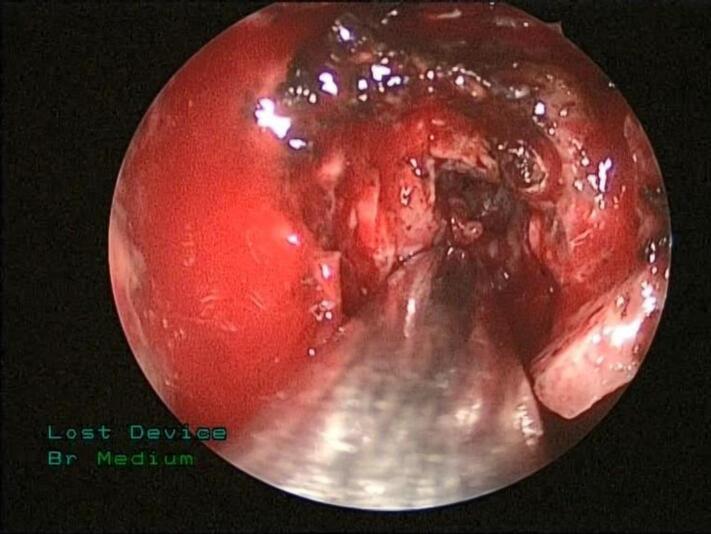
Deep endoscopic tumor removal from the ITF.

The patient underwent a CT-guided biopsy (Fig. 3), which showed clusters of osteoclasts type giant cells with many mononuclear background cells and foamy histiocytes, some of which are hemosiderin laden. The differential diagnosis includes brown tumors of hyperparathyroidism versus GCG. Therefore, the patient underwent endonasal endoscopic trans-maxillary removal of ITF lesion which was performed by an experienced surgeon specialized in rhinology and skull base surgery, the bony posterior wall of the right maxillary sinus was dissected and removed with right ear ventilation tube insertion, as seen in Fig. 4 and Fig. 5. The tumor was dissected from ITF and a biopsy was sent for histopathological analysis, which showed crushed spindle and epithelioid cells with histiocytic differentiation and abundant hemosiderin laden macrophages, admixed with nerves and skeletal muscle fibers. These results confirmed the diagnosis of GCG, and the patient had an uneventful postoperative course and was discharged in a stable condition. The patient had no complications or any sign of recurrence at the one year follow-up.

## 3 Discussion

Giant cell-rich bone lesions represent a diverse group characterized by the proliferation of multinucleated osteoclast-like giant cells. One of these lesions is GCG, an uncommon benign lesion that can occur in both adults and children. GCG is commonly reported in the anterior or posterior mandible and anterior maxilla, though it can also occur in other jaw bones and extragnathic areas [1,4,7]. However, to our knowledge, this is the first reported case of GCG in the ITF.

ITF lesions are rare conditions that usually arise due to tooth extraction, dental infections, fractures involving the maxillary sinus, or infections of the maxillary sinus [8]. Patients with these lesions often present with symptoms such as headache, hearing loss, jaw deviation, auricular/preauricular pain, tinnitus, trismus, or nasal obstruction [9]. Similarly, our patient presented with complaints of decreased hearing, tinnitus, a blockage sensation in the right ear, and jaw claudication on the right side. Although ITF lesions can be caused by benign conditions, they can also signify more serious conditions that require early intervention. Therefore, radiological imaging, including CT and MRI, in addition to clinical assessment, are essential for an accurate diagnosis. CT scan is the preferred initial imaging tool for assessing bone involvement, while MRI plays a critical role in delineating the mass and evaluating its spread around nerves and the brain. According to a previous study, disease spread to the high ITF on CT is associated with poor surgical outcomes and increased morbidity [10].

To reach a provisional diagnosis in cases of ITF lesions, histopathological evaluation must be performed carefully to identify the underlying cause. Both GCG and giant cell tumors can present as ITF lesions, but distinguishing between the two can be challenging due to overlapping histopathological findings. According to a recent study analyzing the histopathological characteristics of the two conditions, giant cells in GCG tend to cluster in groups within the stroma, while in giant cell tumors, they are more uniformly dispersed [11].

## 4 Conclusion

A rare scenario of GCG in the ITF is described in this case report. Imaging and histopathological analysis were crucial for reaching an accurate diagnosis and directing treatment. Early management can effectively avoid complications and recurrence, as evidenced by the successful surgical excision of the lesion using the minimal invasive and direct endonasal endoscopic approach.

Consent.

Written informed consent was obtained from the patient for publication and any accompanying images. A copy of the written consent is available for review by the Editor-in-Chief of this journal on request.

## Ethical approval

This study did not require ethical approval as it is a case report. However, informed consent has been obtained and can be provided on request.

## Sources of funding

This study did not receive any funding.

## Conflict of interest statement

The authors declare that they have no conflict of interests.

## Abbreviations

GCG0: Giant cell granuloma
ITF: Infratemporal fossa
CT: Computed tomography
MRI: Magnetic resonance imaging
